# Evaluation of Nasal Mucociliary Transport Rate by^**99m**^Tc-Macroaggregated Albumin Rhinoscintigraphy in Woodworkers

**DOI:** 10.1155/2011/620482

**Published:** 2011-07-24

**Authors:** Zeki Dostbil, Cahit Polat, İsmail Önder Uysal, Salih Bakır, Askeri Karakuş, Serdar Altındağ

**Affiliations:** ^1^Department of Nuclear Medicine, Dicle University Medical Faculty, 21280 Diyarbakır, Turkey; ^2^Department of Otorhinolaryngology-Head and Neck Surgery, Elazig Research and Training Hospital, 23200 Elazığ, Turkey; ^3^Department of Otorhinolaryngology-Head and Neck Surgery, Cumhuriyet University Medical Faculty, 58140 Sivas, Turkey; ^4^Department of Otorhinolaryngology-Head and Neck Surgery, Dicle University Medical Faculty, 23200 Diyarbakır, Turkey; ^5^Department of Mining Engineering, Dicle University, 21280 Diyarbakır, Turkey

## Abstract

Woodworkers in the furniture industry are exposed to wood dust in their workplaces. The aim of this study is to investigate the effect of occupational wood dust exposure on the nasal mucociliary transport rates (NMTRs) in woodworkers. Twenty five woodworkers and 30 healthy controls were included in this study. Wood dust concentration in workplaces was measured using the sampling device. ^99m^ Tc-macroaggregated albumin (^99m^Tc-MAA) rhinoscintigraphy was performed, and NMTR was calculated in all cases. In statistical analysis, an independent samples *t*-test was used to compare NMTR of woodworkers and control subjects. We found that the mean NMTR of the woodworkers was lower than that of the healthy controls. However, there was not a statistically significant difference between them (*P* = 0.066). In conclusion, our findings suggested that wood dust exposure may not impair nasal mucociliary transport rate in woodworkers employed in joinery workshops.

## 1. Introduction

The term “wood dust” refers to various airborne wood dusts created during the cutting and shaping of softwood, hardwood, hardboard, chipboard, and such other composite materials. Its composition varies depending on the species of tree. Woodworkers in joinery workshops are exposed to wood dust during daily routine work. When workers are working with dust, they should wear protective masks to prevent respirable particle to be inhaled. However, there is usually a lack of protection against inhalable dust particles in the workrooms at industrial estate in Elazığ and Diyarbakır Province, Turkey. Studies on particle size have shown that highest large proportion of airborne wood dust can be trapped in the nasal passage [1, 2]. The EU Directive (1999/38) has classified hardwood dusts as carcinogenic and has set the occupational exposure limit for hardwood dust to 5 mg/m^3^ in workroom air [3].

Mucociliary transport in the nasal cavity is a physiological process in which the mucus layer on the ciliated cells is moving. It is an important defense mechanism against physical and biological insult in the nasal fossa, paranasal sinuses, and lower respiratory tracts. Inhaled foreign particles and micro-organisms are caught by the mucus and transported towards the nasopharynx by means of nasal mucociliary activity (MCA). This process has a protective effect on the upper and lower respiratory system and is considered a first-line defense mechanism in humans. This effect depends on factors including the number of cilia and beat frequency, their coordinated movements, the amount of nasal fluid, and its viscoelastic properties [4]. If this function is impaired, the protective effect of nasal cilia may be lost.

Nasal complaints have been reported in woodworkers exposed to wood dust [5–7]. The impairment of nasal mucociliary clearance, together with nasal symptoms in woodwork teachers, was previously reported by some researchers [8, 9]. Impaired mucociliary clearance has been reported as a mechanism for nasal complaints in relation to environmental wood dust [5, 10]. In literature, studies on the relationship between occupational wood dust exposure and nasal mucociliary transport rates (NMTRs) are scarce.

In this study, we aimed to investigate the effect of occupational wood dust exposure on nasal NMTR in woodworkers using ^99m^Tc-MAA rhinoscintigraphy.

## 2. Materials and Methods

Twenty five woodworkers in joinery workshops at an industrial estate in Elazığ and Diyarbakır Province, Turkey, were included in this study. They used mainly pine wood, chipboards, medium-density fiberboards, and hardwoods in their routine work. All of them worked at or near the sawmill machine. All sawmill machine had their own dust collecting system. None of workers used protective mask. All of them breathed in the fine dust particles during all day. All of them had been already working in their jobs. In the control group there were 30 healthy individuals, none of whom had previously been exposed to intense environmental dusts. Exclusion criteria were cigarette smoking, chronic drug use, a history of upper respiratory tract infection in past two weeks, allergic rhinitis, chronic nasal obstruction, chronic or current nasal drainage, nasal septal deviation, nasal polyposis, or a history of any chronic disease. Before starting the study, we obtained ethical approval from the Elazığ Training and Research Hospital Ethics Committee, and written consent was given by all study subjects. All subjects were examined by an ear, nose, and throat specialist and were excluded from the study if any abnormal findings were determined during the ear, nose, and throat or head and neck examinations. The time elapsed between the last wood dust exposure and the scintigraphic study of the woodworkers was between two and five hours. Rhinoscintigraphy was performed by dripping one droplet (~50 *μ*Ci that corresponds to about 25 *μ*Sv radiation exposure) of ^99m^Tc-MAA (particle size ranged between 10 and 150 *μ*m) on right side, on base of the nasal meatus and the anterior end of the inferior turbinate by using a 27 G syringe. A scintigraphic procedure gives study subjects only a negligible Gamma radiation exposure as a very small dosage of   ^99m^Tc-MAA is used. Room temperature was stabilized at 21°C. In the supine position, images were obtained by using a GE-Infinia and GE-millennium *γ* camera system (GE Medical Systems, Milwaukee, WI, USA) with an LEHR collimator and detectors were set laterally. Thirty-second dynamic images were obtained for a period of 20 minutes. After the test, the images were processed to determine NMTR in millimeters per minute (mm/min). The distance between the point where the radiopharmaceutical was dropped and the point where the particles reached the nasopharynx cavitiy was measured on a straight line using a system computer. Then, to determine the NMTR in mm/min, this length was divided by the time elapsed.

### 2.1. Wood Dust Measurement

Wood dust samples were collected by an automatic isokinetic sampling device (Isostack Basic, TCR TECORA, Milan, Italy). This machine was placed in seven workplaces used by the woodworkers. Wood dust particles less than 10 *μ*m in size were regarded as respirable. A certain amount of the room air was collected automatically by using a PM10 head that prevents the collection of wood dust particles larger than 10 *μ*m for a certain time. Then the filters placed inside the device were removed and the trapped wood dust particles collected and measured in mg/m^3^ using a standard procedure.

### 2.2. Statistical Analyses

An independent samples *t*-test in the SPSS 15.0 statistical program was used to compare NMTR of the controls and the woodworkers. Pearson's correlation coefficient was employed in the correlation analyses, with the results taken to be statistically significant when *P* < 0.05.

## 3. Results

**Figure 1:**
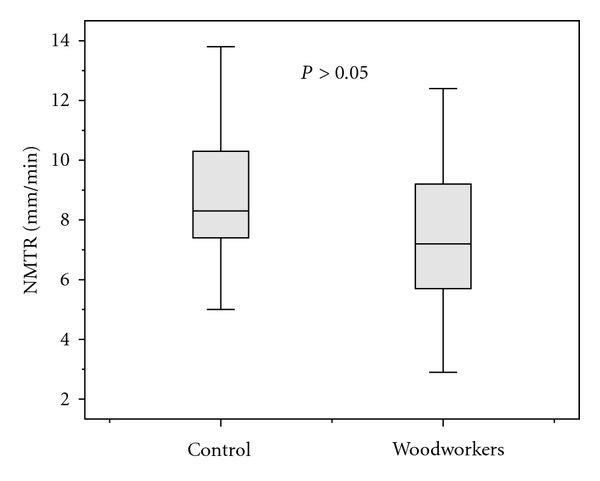
Nasal mocociliary transport rates in woodworkers and control subjects.

All subjects were male and the mean age of the woodworkers was 31.3 ± 10.4 years (range, 19–54 years). The mean age of the control subjects was 27.6 ± 8.7 years (range, 15–47 years). Exposure duration, daily exposure, and respirable wood dust concentration of workplaces were shown in Table 1. Significant difference was not found between ages of the control subjects and the workers (*P* = 0.155). The mean NMTR value of woodworkers was 7.5 ± 2.4 mm/min (range, 2.9–12.4 mm/min) and, the mean NMTR of healthy controls was 8.7 ± 2.3 mm/min (range, 5–13.8 mm/min). There was not statistically significant correlation between NMTR and the age of workers, exposure duration, daily exposure, and wood dust concentrations in workshops (*P* > 0.05; *r* = −0.03, *r* = −0.13, *r* = −0.21, and *r* = −0.05, resp.). The mean NMTR of the woodworkers was lower than that of the healthy controls (Figure 1). But, statistical analysis revealed that the mean NMTR value for woodworkers was not significantly lower than that of the healthy controls (*P* = 0.066).

## 4. Discussion

In woodworkers exposed to occupational wood dust, it is possible to see an increased risk of asthmatic symptoms [7, 11, 12], increased frequency of chronic bronchitis [8], nasal symptoms [5, 8, 13], eye symptoms [8], decreased baseline lung function [11], and a decline in lung function. Furthermore, many researchers reported that the wood dust exposure may increase the cancer risk [7, 14]. Dahlqvist et al. [15] reported acute effects in healthy volunteers caused by the exposure to air contaminants in a sawmill. In that study, the volunteers displayed a slight inflammatory reaction of the upper airways. The importance of decreasing the concentration of particles in the working environment was noted.

In our study, we found that the mean NMTR of woodworkers was lower than that of healthy controls (7.5 ± 2.4 mm/min; 8.7 ± 2.3 mm/min, resp.). However, statistical analysis has shown that this difference was not statistically significant (*P* = 0.066). This finding demonstrates that wood dust exposure at a mean concentration of 1.9 mg/m^3^, which is lower than the EU limit for hardwood of 5 mg/m^3^, does not significantly impair nasal NMTRs of woodworkers. 

Black et al. [10] studied mucociliary clearance by using 5 *μ*m ^99m^Tc-particles in nine woodworkers in the furniture industry. They reported that the clearance of radiolabeled particles was generally much slower in the woodworkers than that in the controls. In that study, they concluded that this limited investigation of exposure to wood dust in the furniture industry for ten years or more could impair nasal mucociliary clearance. Their findings are not consistent with our results. Five of nine woodworkers enrolled in their study were smokers, and a detailed ear, nose, and throat examination had not been performed by a specialist. However, it has been reported in some studies that smoking, and other certain conditions such as nasal septal deviation and nasal polyposis, may impair mucociliary clearance [16–18]. Moreover, the Black et al. study does not include statistical analyses, probably because of the limited patient numbers. In their report, they stated that a more detailed study would be required as it was performed on a very limited number of subjects. In our study, we excluded the subjects having conditions that are known to impair nasal MCA such as cigarette smoking, allergic rhinitis, nasal septal deviation, and nasal polyposis. Although the NMTR of woodworkers was lower than that of controls (7.5 ± 2.4 mm/min; 8.7 ± 2.3 mm/min, resp.), statistical analysis did not show any statistically significant difference between NMTRs of woodworkers and control subjects (*P* = 0.066).

Wilhelmsson and Drettner [5] studied nasal problems in wood furniture workers. They measured nasal mucociliary clearance by using a saccharine test in 61 woodworkers and did not find any significant difference between mucociliary clearance rates of workers and the control group. Our findings were consistent with their results. In the saccharin test, a saccharin solution is dropped in the nasal cavity. This substance is carried to the nasopharynx and causes the patient to sense a taste of sugar. The time interval between the dropping process and sensing the sugar taste is noted. The main disadvantages of this test are that the NMTR cannot be measured, and it relies on the patient's sense of taste. Rhinoscintigraphy can supply objective and detailed information that allows for quantitative analyses [19]. It has been shown to be a reliable, easily reproducible, and harmless method for measurement of nasal MCA. Currently most researchers use ^99m^Tc-MAA for rhinoscintigraphy [20]. For this reason, we also preferred ^99m^Tc-MAA rhinoscintigraphy for the measurement of nasal MCA in our study. 

Our study does have some limitations. We did not detect number of cilia per unit area or cilia beat frequency by using other methods to reveal whether any mucosal damage had been occurred. Nevertheless, our study has shown that nasal mucociliary function worked properly in the woodworkers. We measured wood dust concentration in a short space of time, which may not be representative of the long-term exposure by workers. 

In conclusion, our study findings have shown that occupationally wood dust exposure in joinery workshops may not impair nasal mucociliary transport rate in woodworkers. But this does not mean that it does not have any hazardous effects on respiratory system.

## Figures and Tables

**Table 1:** Some parameters of the woodworkers and the control subjects (mean ± SD and range).

	NMTR	Age	Exposure duration	Daily exposure	Respirable wood dust
	(mm/min)	(year)	(year)	(hour)	(<10 *μ*m) (mg/m^3^)
Woodworkers *n* = 25	7.5 ± 2.4	31.3 ± 10.4	16.4 ± 11.3	9.7 ± 1.3	1.9 ± 0.3
	(2.9–12.4)	(19–54)	(3–44)	(8–12)	(1.3–2.4)
Control *n* = 30	8.7 ± 2.3	27.6 ± 8.7			
	(5–13.8)	(15–47)			

NMTR: nasal mucociliary transport rate.
